# Data fusion and 3D visualization for optimized representation of neurovascular relationships in the posterior fossa

**DOI:** 10.1007/s00701-021-05099-1

**Published:** 2022-01-11

**Authors:** Peter Hastreiter, Barbara Bischoff, Rudolf Fahlbusch, Arnd Doerfler, Michael Buchfelder, Ramin Naraghi

**Affiliations:** 1 Department of Neurosurgery, Friedrich-Alexander University Erlangen-Nuremberg, Schwabachanlage 6, 91054 Erlangen, Germany; 2 Department of Neurosurgery, St. Marien Hospital, Amberg, Germany; 3 International Neuroscience Institute, Hannover, Germany; 4 Division of Neuroradiology, Friedrich-Alexander University Erlangen-Nuremberg, Erlangen, Germany; 5 Department of Neurosurgery, Central Hospital of German Armed Forces, Koblenz, Germany

**Keywords:** Neurovascular relationships, Posterior fossa, Data fusion, 3D visualization, Compression syndromes

## Abstract

**Background:**

Reliable 3D visualization of neurovascular relationships in the posterior fossa at the surface of the brainstem is still critical due to artifacts of imaging. To assess neurovascular compression syndromes more reliably, a new approach of 3D visualization based on registration and fusion of high-resolution MR data is presented.

**Methods:**

A total of 80 patients received MRI data with 3D-CISS and 3D-TOF at 3.0 Tesla. After registration and subsequent segmentation, the vascular information of the TOF data was fused into the CISS data. Two 3D visualizations were created for each patient, one before and one after fusion, which were verified with the intraoperative situation during microvascular decompression (MVD). The reproduction quality of vessels was evaluated with a rating system.

**Results:**

In all cases, the presented approach compensated for typical limitations in the 3D visualization of neurovascular compression such as the partial or complete suppression of larger vessels, suppression of smaller vessels at the CSF margin, and artifacts from heart pulsation. In more than 95% of the cases of hemifacial spasm and glossopharyngeal neuralgia, accurate assessment of the compression was only possible after registration and fusion. In more than 50% of the cases with trigeminal neuralgia, the presented approach was crucial to finding the actually offending vessel.

**Conclusions:**

3D visualization of fused image data allows for a more complete representation of the vessel-nerve situation. The results from this approach are reproducible and the assessment of neurovascular compression is safer. It is a powerful tool for planning MVD.

## Introduction

The understanding of the complex nerve-vessel relationships in the posterior fossa on the surface of the brainstem is of high importance for the assessment of neurovascular compression (NVC) syndromes [13, 15]. Established sequences for the assessment of NVC are high-spatial-resolution strong 3D gradient echo T2-weighted imaging, i.e., Constructive Interference in Steady State (CISS), and MR-angiography, i.e., Time-of-Flight (TOF) [14, 19, 22]. In addition to that, the higher signal of 3.0 Tesla can improve the spatial resolution for the assessment of NVC [7].

The introduction of a standardized image processing and visualization method has proven to be a powerful tool for analyzing neurovascular relationships [10, 11, 16, 23]. As an important advantage of this strategy, the nerve and vessel structures are effectively delineated with implicit segmentation based on transfer functions for color and opacity values after explicit segmentation of coarse structures in the beginning.

Major problems limiting the quality of 3D-visualizations are the flow-related signal variability of vessels and pulsation artifacts of the cerebrospinal fluid (CSF) [16]. This leads to a considerably inferior representation of larger vessels, e.g., basilar artery (BA), vertebral artery (VA), and related vascular junctions in CISS data. In addition, vessels at the boundary of the CSF suffer from contour fusion with neighboring structures, thereby preventing their precise delineation [16]. On the other hand, in-plane and small vessels are not consistently reproduced in TOF data due to known restrictions [7]. To solve these problems, the principles of data fusion based on the source data are applied for the first time and the corresponding results of the 3D visualization are presented. Some studies claim to perform image fusion, but limit themselves to combining rendering results of different image data. In contrast, to solve the problems described above, the principles of the fusion of source data are described for the first time and the results of the 3D visualization are evaluated.

## Material and methods

### Patients

In a prospective study, a total of 80 patients (f:m, 46:34) with trigeminal neuralgia (TN, *n* = 54), hemifacial spasm (HFS, *n* = 22), and glossopharyngeal neuralgia (GN, *n* = 4) were considered in this work. After preoperative diagnostics, microvascular decompression (MVD) was performed on all patients. The ethics committee of the University approved this study. Informed consent was obtained from all patients included in the study.

### Image data

All patients obtained image data with a 3.0 Tesla MR system (Magnetom Trio, Siemens Healthcare, Erlangen, Germany). The two sequences CISS and TOF were used. CISS provides images with a high degree of contrast between the hyperintense cerebrospinal fluid (CSF) and the hypointense cranial nerves, brainstem, and vessels. TOF is the most commonly used MR angiography sequence in neurovascular imaging with the highest resolution. Using the signals of floating fluid, it displays the vessels as hyperintense structures without the use of a contrast agent [18]. Both sequences were adjusted to isotropic voxel size (0.4 mm) ensuring equally high resolution in axial, coronal, and sagittal slice images. Details of the sequence parameters were previously published [7, 16].

### Registration

**Fig. 1:**
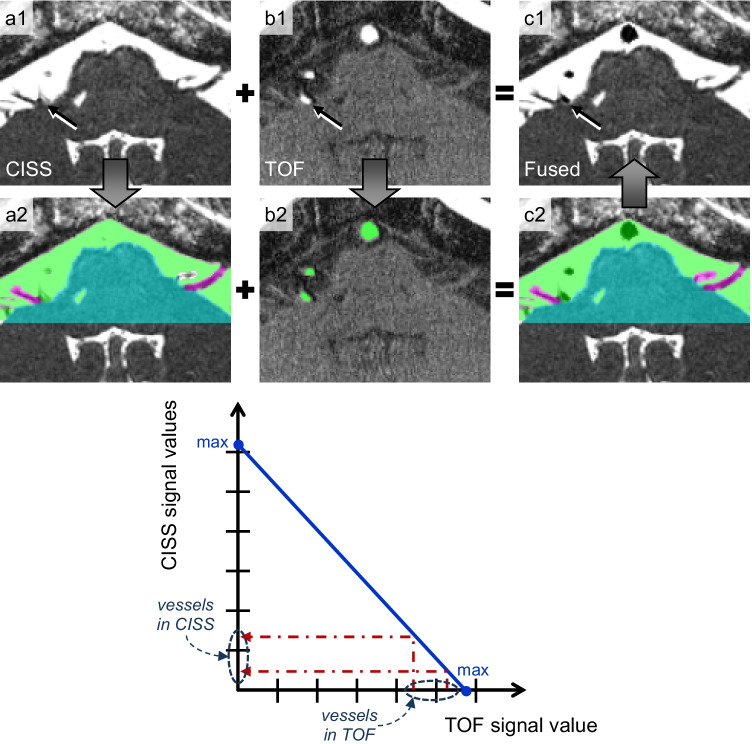
Innovative fusion process: fused slice image (c1) in hemifacial spasm after registration of CISS (a1) and TOF (b1) showing the compression site (arrow) and the improvement of the limited vascular presentation in CISS by supplementing TOF information. The segmentation images demonstrate for CISS (a2) the separation of brainstem, nerves, and CSF including all vessels, for TOF (b2) the separation of vascular structures and the fused segmentations of CISS and TOF (c2). The function shows how the signal values of the segmented voxels with vessel information in the TOF data were inverted to match the range of values of the CISS data. To determine this function, the maximum signal values in the CISS and TOF data were determined and a line equation was thus established

To improve the representation of the vessels in the 3D visualization, CISS and TOF data were registered. Initial alignment was achieved during imaging by performing both sequences immediately after each other. In order to compensate for motion artifacts and to obtain accurate registration of the data, an automatic entropy-based approach using a rigid transformation was used [24]. After reformatting the TOF data according to the grid of the CISS data, the arrangement, number, and size of the voxels in both data became identical. Figure 1 shows corresponding slices after registration.

### Segmentation of CISS data

In CISS data, cranial nerves and vessels are represented in the same range of intensity values as the surrounding tissues. For a clear 3D visualization, an explicit segmentation of the CISS data is mandatory as presented previously [10, 16]. In a first step, the CSF area of the basal cisterns ventral and lateral to the brainstem was semi-automatically segmented with volume growing. An upper and lower threshold served to limit the range of intensity values and a bounding box helped to restrict the volume of interest. In the next step, the brainstem was interactively delineated. The segmentation to the CSF area, which was precisely determined in the preceding step, was defined as constant. This served as a major support of rapid processing. Then, the cranial nerves were segmented by manual labeling. As a result, the CISS data is divided into four sub-volumes according to the respective anatomical structures: (1) CSF area including all vascular structures, (2) brainstem, (3) cranial nerves, and (4) all remaining structures.

### Segmentation of TOF data

For the TOF data, volume growing was applied to extract the vascular structures. Since vessels in TOF data have higher intensity values than the other tissues, a lower threshold was sufficient to limit the growing process. A bounding box was used to restrict the procedure to the area of interest. Finally, the segmented vessels in the TOF data were labeled with the same tag as the vessels in the CISS data, i.e., identical to sub-volume 1 (CSF region including all vascular structures).

### Fusion of CISS and TOF data

The basis of the presented fusion strategy is a direct correspondence of pairs of voxels in the CISS and TOF data, which is ensured by reformatting the TOF data in the grid of the CISS data following registration. As a further prerequisite for fusion, the segmentation of both the CISS and TOF data assigns an identical label to all voxels that describe vessels. In order to match the values of the labeled voxels, the hypertensive values of the voxels marked as vessels in TOF data were inverted and mapped into the hypointense value range of vessels in the CISS data (see Fig. 1). For this inversion, the maximum voxel values in the CISS and TOF data were determined and thus an equation of a line was established. The linear mapping thus described was used to convert the values of all labeled voxels in the TOF data into the value range of the CISS data. Thereafter, for each pair of corresponding voxels, the original CISS values were replaced by the inverted TOF values. As a result, the vascular information of both data was combined and a new dataset with fused TOF and CISS information was obtained (see Fig. 1).

### 3D visualization

For the 3D visualization, an implementation for direct volume rendering (dVR) was used, which allows using an individual transfer function for every sub-volume [11, 12, 16]. Thereby, the data values of the four sub-volumes are mapped to different color and opacity values. The following colors were used: red for vessels, yellow for nerves, and light gray for brainstem. All remaining structures (sub-volume 4), which do not provide relevant information for NVC syndromes, were made completely transparent.

All data was visualized both with 2D visualization using slice images and with 3D visualization using direct volume rendering before and after fusion. In this work, the following terms are used for 2D visualizations with slice images: 2D-vis-CISS and 2D-vis-FUSION, based on CISS data only and fused CISS and TOF data, respectively. Similarly, for 3D visualizations with direct volume rendering, the following terms are used: 3D-vis-CISS and 3D-vis-FUSION based on CISS data only and fused CISS and TOF data, respectively.

### Evaluation of the fused data

**Fig. 2:**
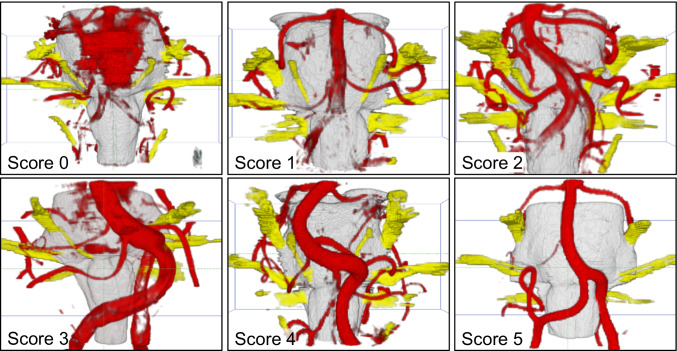
Assessment scheme for the evaluation of the visualizations. Score 0: Missing representation of the vessel. (Here: The vertebral artery is missing. The basilar artery and the superior cerebellar artery are completely covered by pulsation artifacts). Score 1: The vessel can be guessed, but no statements about the anatomical facts are possible (Here: The vertebral artery is only schematically shown). Score 2: Important parts of the vessel are missing and only peripheral segments can be seen. (Here: The vertebral artery can be recognized, but some parts are missing). Score 3: The proximal parts of the vessel are shown (Here: Only the proximal parts of the vertebral artery are visible). Score 4: Relevant parts of the vessel, mainly related to the compression area, are visualized (Here: Important parts of the vertebral artery are present and allow reconstructing the course of the vessel). Score 5: Complete representation of the vessels and their branches

Two 3D visualizations were created for each patient, one before and one after fusion. All were interactively compared and verified with the intraoperative situation during microvascular decompression (MVD). A rating system developed by our working group was used to assess the visualization quality of the vessels (see Fig. 2). Two independent expert observers (neurosurgeon with regular experience in MVD) made the evaluation.

Particular attention was paid to the effects of artifacts since they can significantly influence the visualization. Flow-related artifacts are common in vessels with a wide lumen. In general, moving spins do not contribute to the signal formation of a given voxel at MRI and result in vessels not being displayed [18]. Artifacts by pulsation are motion artifacts caused by CSF pulsation and blood flow. In the area of the posterior fossa, especially the basilar artery vibrates in the CSF due to the pulse wave. In the visualization, this appears as a foggy cloud. Finally, contour fusion is caused by similar intensity values of vessels and brainstem in the CISS data [16]. If the course of a vessel is on the surface of the brainstem, the segmentation can lead to an incorrect labeling and thus to a vessel, which in the visualization becomes brainstem.

## Results

**Fig. 3:**
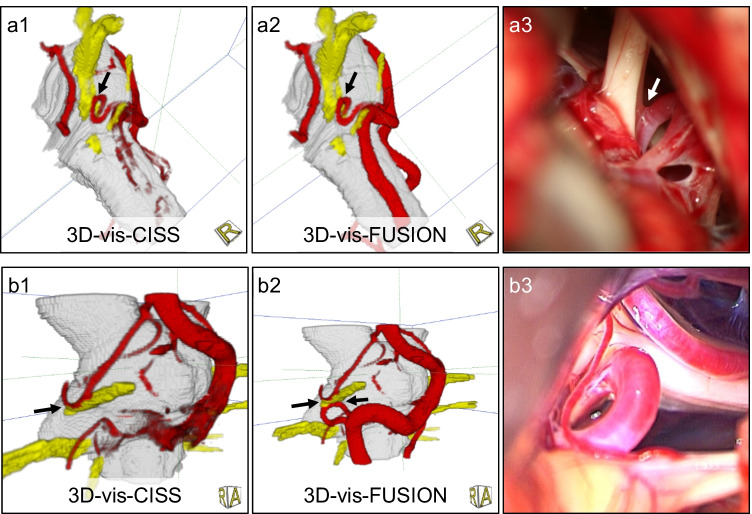
Innovative 3D visualization represents real anatomy. A case of hemifacial spasm (a1-3) with scores before fusion (a1: PICA = 5, VA = 2) and scores after fusion (a2: PICA = VA = 5). A case of trigeminal neuralgia with sandwich compression (b1-3), with scores before fusion (b1: SCA = 5, AICA = 0, VA = 1) and with scores after fusion (b2: SCA = AICA = VA = 5). It shows incomplete representation of the vessels with CISS only (a1, b1) and clear representation of the nerve-vessel relationship and NVC (arrow) with fused data (a2, b2) according to the intraoperative situation (a3, b3)

The presented approach was successfully applied in 80 consecutive cases of NVC syndromes for preoperative analysis. To evaluate the improvements achieved by fusion, all visualizations (for each case: 2D and 3D before and after fusion) were evaluated using the scheme of Fig. 2 and compared to the intraoperative situation during MVD (see Fig. 3).

The results presented in Table 1 demonstrate that the suggested fusion method leads to considerably improved 3D visualization and slightly improved 2D visualization. With regard to the applied assessment scheme, the total score for 3D visualization was improved by more than one level so that the important nerve and vessel structures are presented. A more detailed analysis of Table 1 shows that the smaller vessels (i.e., AICA and SCA) are best represented in 2D-VIS-CISS, whereas the larger vessels (i.e., A. basilaris and A. vertebralis) were usually shown only in a schematic way. In contrast, the slice images of TOF mainly showed the larger vessels (i.e., A. basilaris and A. vertebralis) in an optimal way. After fusion, 3D-vis-FUSION improved the score of the larger vessels by 1 to 2 points compared to 3D-vis-CISS. On the other hand, the smaller vessels after fusion were only marginally better represented (i.e., AICA: improved by an average of 0.45 points, SCA: improved by an average of 0.8 points).

**Table 1:** Data evaluation: mean values (m) and standard deviations (σ) of the vessel representation in the 2D and 3D visualizations of 80 cases according to the 0 to 5 scoring scheme in Fig. 2. For each case, the visualizations were evaluated before (2D- and 3D-vis-CISS) and after (2D- and 3D-vis-FUSION) the fusion

*n* = 80	2D visualization (slices)		3D visualization	
	2D-VIS-CISS	2D-VIS-FUSION	3D-vis-CISS	3D-vis-FUSION
Vessel	m (σ)	m (σ)	m (σ)	m (σ)
A. basilaris	3.62 (1.65)	4.93 (0.47)	3.40 (1.76)	4.80 (0.78)
A. vertebralis ri	3.66 (1.57)	4.59 (0.85)	2.13 (1.49)	4.49 (0.93)
A. vertebralis le	3.57 (1.53)	4.60 (0.82)	2.18 (1.49)	4.52 (0.85)
PICA ri	2.93 (2.05)	3.53 (2.11)	2.19 (1.87)	3.60 (1.96)
PICA le	2.70 (2.01)	3.68 (2.05)	1.82 (1.59)	3.72 (1.88)
AICA ri	4.00 (1.54)	3.04 (1.90)	3.06 (1.79)	3.59 (1.74)
AICA le	3.58 (1.77)	2.42 (2.01)	2.75 (1.97)	3.11 (1.95)
SCA ri	3.89 (1.56)	3.98 (1.56)	2.92 (1.66)	3.66 (1.55)
SCA le	3.87 (1.49)	4.03 (1.61)	2.89 (1.65)	3.75 (1.59)
**Total**	**3.60 (1.70)**	**3.88 (1.74)**	**2.65 (1.77)**	**3.96 (1.59)**

**Fig. 4:**
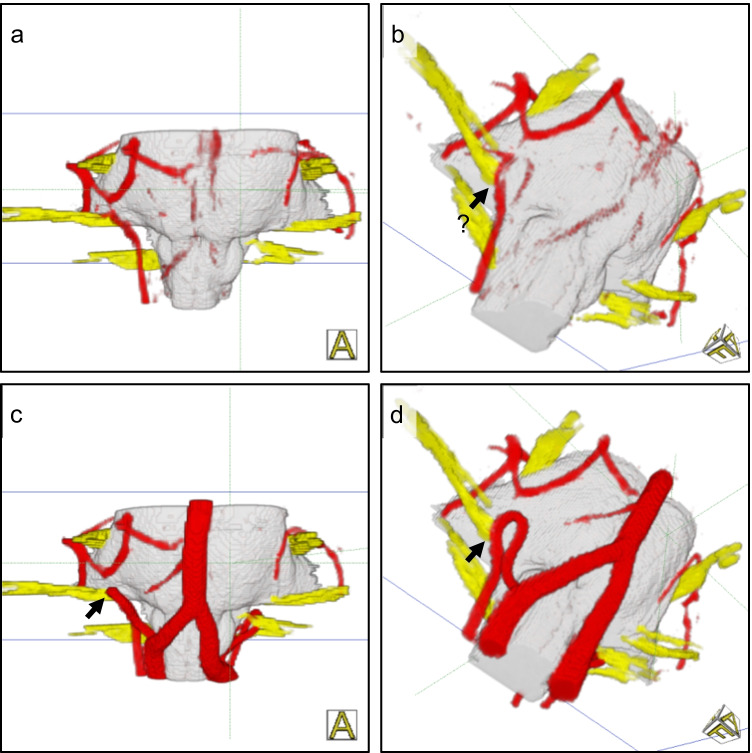
3D visualization of a right-sided hemifacial spasm: Before fusion, important vessels are not shown (a, b) with scores BA = VA = 0 and PICA = 1. After fusion, the vessel-nerve contact (arrow) is clearly presented (c, d) with scores BA = VA = PICA = 5

**Fig. 5:**
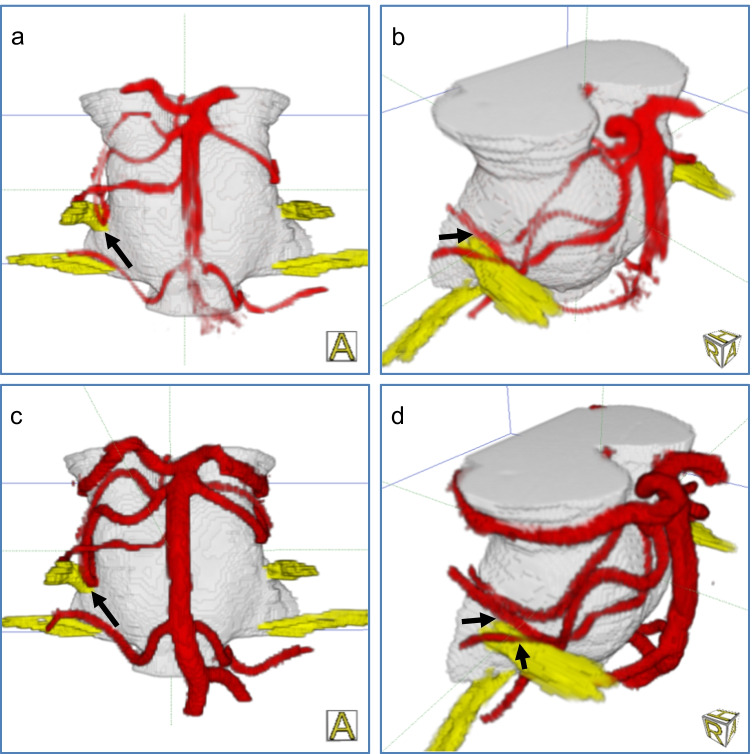
3D visualization of a right-sided trigeminal neuralgia: Before fusion, the vessel-nerve contact (arrow) is only imprecisely identified (a, b) with scores BA = AICA = SCA = 4, vein = 3). After fusion, the compression site (arrow) is safely analyzed (c, d) with scores BA = AICA = SCA = vein = 5)

The improved representation of NVC syndromes by the fusion of CISS and TOF data is illustrated in Fig. 4 for a case of hemifacial spasm and in Fig. 5 for a case of trigeminal neuralgia.

**Fig. 6:**
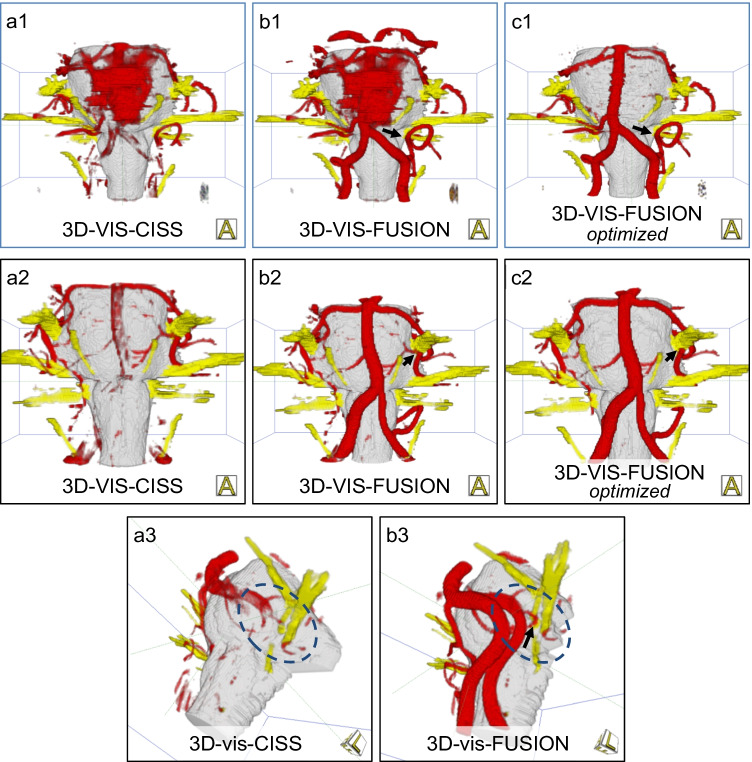
Reduction of artifacts. Pulsation artifacts (top row) in the rostral part around the basilar artery and flow related artifacts in the caudal portion around the vertebral artery and branches in a case of hemifacial spasm caused by the left PICA (a1). Fusion completes the vessels and shows the site of compression (arrow), but does not reduce the artifacts (b1). Subsequent optimization of the segmentation leads to complete suppression of the artifacts (c1). Flow artifacts (middle row) in a case of trigeminal neuralgia caused by the SCA and a vein on the left: the vertebral arteries are not present and the basilar artery is only schematically shown (a2). Only after fusion do the basilar and vertebral arteries clearly appear (b2) and the site of compression is clearly assigned (arrow). Optimization of the segmentation does not provide further improvement (c2). Contour fusion (bottom row): in a case of hemifacial spasm, the PICA, causing the NVC (arrow), is extinguished before fusion (a3) and presented after fusion (b3). The missing vertebral arteries are related to flow artifacts (a3)

Pulsation artifacts could not be avoided despite absolute resting position of the patient during the MRI measurement. They can completely or partially obscure relevant vessels (see Table 2). As shown in Fig. 6, the assessment of the vessel-nerve relationships in the 3D visualizations of CISS data (3D-vis-CISS) was significantly affected. The proposed fusion method helped to complement the vessels in the 3D visualization of the fused data (3D-vis-FUSION), but reduced the artifacts only to a limited extent. If the underlying segmentation was further optimized through expert knowledge, the number of artifacts could be significantly reduced (3D-vis-FUSION optimized). The improvement was less effective and much more time-consuming if the optimization was attempted only with CISS data and without fusion; see Table 2.

**Table 2:** Reduction of artifacts: Starting from the 3D visualization of the CISS data (3D-vis-CISS), improvement through optimization with expert knowledge (optimized) as well as through image fusion (3D-vis-FUSION) and subsequent optimization with expert knowledge (optimized)

*n* = 80	**Pulsation artifacts**			
**Vessel**	**3D-vis-CISS**	**3D-vis-CISS** *optimized*	**3D-vis-FUSION**	**3D-vis-FUSION** *optimized*
A. basilaris	41	6	38	1
A. vertebralis ri	22	6	16	1
A. vertebralis le	22	6	19	1
PICA ri	13	0	10	0
PICA le	16	0	12	0
AICA ri	0	0	0	0
AICA le	0	0	0	0
SCA ri	10	3	10	0
SCA le	10	3	10	0
*n* = 80	**Flow artifacts**			
**Vessel**	**3D-vis-CISS**	**3D-vis-CISS** *optimized*	**3D-vis-FUSION**	**3D-vis-FUSION** *optimized*
A. basilaris	51	48	0	0
A. vertebralis ri	70	67	0	0
A. vertebralis le	70	70	0	0
*n* = 80	**Contour fusion artifacts**			
**Vessel**	**3D-vis-CISS**	**3D-vis-CISS** *optimized*	**3D-vis-FUSION**	**3D-vis-FUSION** *optimized*
PICA ri	11	10	4	0
PICA le	11	9	5	0
AICA ri	15	14	4	1
AICA le	19	17	7	0
SCA ri	5	4	1	1
SCA le	5	5	2	0

Flow artifacts were particularly noticeable in the larger vessels, while they occurred less with smaller vessels. In particular, the basilar and the vertebral artery were strongly affected in the 3D visualization of the CISS data (3D-vis-CISS), as shown in Fig. 6. Their course could not be fully represented in 65–90% of the cases and vessel junctions could not be objectively understood. The quantitative evaluation in Table 2 shows that the problem could not be solved if the segmentation of only the CISS data had been optimized (3D-vis-CISS optimized). In contrast, all flow artifacts were effectively eliminated by fusion (3D-vis-FUSION). If the segmentation of the fused data was further optimized, the resulting visualization (3D-vis-FUSION optimized) was slightly better but it did not substantially improve the analysis of the NVC.

Contour fusion was mainly observed in the smaller vessels and with venous structures. As Table 2 shows, it was observed mainly in the 3D visualization of the CISS data (3D-vis-CISS) in the PICA and AICA and less in the SCA. The affected vessel was interrupted or completely extinguished due to incorrect segmentation (see Fig. 6). With the proposed fusion approach, the problem could be significantly improved (3D-vis-FUS). In consequence, the segmentation is more robust because the inversion of the TOF signal values suppresses the noise in the area of the vessels and allows distinguishing the values of vessels and brainstem. If the segmentation of the fused data was additionally optimized (3D-vis-FUS optimized), it was possible to achieve further improvement.

The processing of the CISS data was straightforward and followed the established procedure previously described by the authors [10, 16]. The segmentation of TOF data was unproblematic due to the clear signal intensities of vessels. Only if the noise level was higher, the separation of smaller vessels was restricted in 8 cases. The missing information could be compensated in these cases by the information in the CISS data.

Using the suggested fusion approach, vessels are represented with a clearer signal intensity that has in consequence simplified segmentation in all cases. The time originally required for image processing was ranging between 2 to 4 h [16]. With the proposed fusion approach, it was reduced by 60% to an average of 1 h.

The comparison with the intraoperative situation during MVD revealed that an accurate and safe assessment of NVC was possible in more than 95% of the cases of hemifacial spasm and glossopharyngeal neuralgia (see Fig. 3). In more than 50% of the cases with trigeminal neuralgia, the presented approach was crucial to finding the actually offending vessel.

## Discussion

The exact understanding of the relationship of cranial nerves and vascular structures in the posterior fossa at the surface of the brainstem is crucial for preoperative analysis of NVC. This study presents an approach of 3D visualization based on registration and fusion of high-resolution CISS and TOF data, effectively complementing the authors’ established visualization method [10, 11, 16]. In contrast to all previous studies, the presented work quantitatively analyzed data obtained with MR imaging and image processing by comparing 3D-visualization of CISS data (3D-vis-CISS) and 3D-visualization of fused CISS and TOF data (3D-vis-FUSION). Overall, the presented approach leads to a significantly improved assessment of NVC and makes the evaluation of NVC faster and safer.

When the technical fundaments of interactive and virtual 3D visualization of NVC syndromes were introduced [1, 2, 16], it was proposed almost parallel to register and fuse additional vascular information from angiographic data [10, 11]. As described in further studies, MR angiography data can effectively complement the information gained from strongly T2-weighted MR data. In this context, Zeng et al. preoperatively analyzed the neurovascular relationships in TN using 2D visualization based on slice images without registration and fusion [27]. Haller et al. presented a study on imaging of NVC syndromes, in which fused CISS and TOF data with 2D-visualizations using slice images were presented without information about the fusion strategy used [9]. Docampo et al. used similar data at 3.0 Tesla, visualized the TOF data with volume rendering and transferred the resulting 3D visualization into slice images to investigate NVC in TN [4]. The technique of fusion is not specified and the subsequent result is not a complete 3D representation. Yao et al. used data primarily at 1.5 Tesla to visualize them in 3D with surface rendering. The information of the different data was only fused in the 3D visualization by selecting the modality that offers the highest contrast of the respective target tissue [26]. In this strategy, major information of structures within the CSF cisterns was not taken into account during image processing and was therefore not provided in the resulting 3D representations. A large proportion of the studies does not refer to the applied methods of image processing, which are, however, of fundamental importance for understanding and interpreting of the created 3D visualization.

Further studies about fusion at NVC have considered image data from different sequences or modalities and different visualization techniques. Oishi et al. used high-resolution MR and CT data to generate interactive 3D representations to prepare MVD [17]. Guo et al. combined different T1- and T2-weighted MR data to study neurovascular compression in facial neuralgia [8], and El Refaee et al. compared the MR data with intraoperative high definition endoscopic visualization in HFS [6].

Other studies have correlated the results of imaging and image processing with surgical findings in order to analyze NVC in TN, HFS, and GN [13, 14, 21]. It turned out that 2D-representations do not give sufficient overview of the spatial relationships of nerves and vessels.

There is general agreement that high-resolution sequences are necessary tools to assess the neurovascular relationships in NVC correctly. The quality of the source data is of paramount importance for the quality of the 3D visualization [16, 25]. There is further consensus that patients with NVC may benefit from higher resolution and greater sensitivity of 3.0 Tesla MRI [7]. As a limitation, we observed high signal intensities in the center of the lumen within the basilar artery and vertebral arteries in almost every CISS data, significantly affecting 3D visualization. In 25 cases, the contrast between vessels and CSF was completely lost (see Fig. 1). In consequence, the vessel could not be presented in 3D visualization.

An approach that combines the original source data in such a way that a new dataset is created with fused source information has not been proposed until now. In the presented approach, the original CISS and TOF data are fused on the level of the voxels and a fused dataset is generated which is then used for 3D visualization. As a major advantage of this strategy, only that information is taken from the data that optimally represent a structure or part of it. In this way, the smaller vessels (i.e., AICA and SCA) are primarily taken from the CISS data and the larger vessels (i.e., A. basilaris and A. vertebralis) more from the TOF data. The decision as to which vessel to take from which data is automatically controlled by the segmentation of the CISS and TOF data as well as the technique for inverting the TOF signal values.

In the present work, the quality of the 3D visualizations was assessed objectively by evaluating the imaging and reproduction of the vessels before and after fusion. Our data show that the suggested fusion of CISS and TOF data leads to a reliable 3D representation of the cranial nerves and at the same time to a significantly increased quality of vessel presentation. Thereby, an accurate and reliable identification of the nerve-vessel relationships is available (see Fig. 3, Fig. 4, Fig. 5).

We were able to assess a 49% increase in the quality of the vessel presentation for 3D-vis-FUSION compared to 3D-vis-CISS. A detailed analysis showed that the mean display quality was higher for all vessels in 3D-vis-FUSION than in 3D-vis-CISS. Adequate reproduction of vessels in 3D-visualizations has shown to be crucial for the detection of NVC. Poor vessel reproduction due to flow and pulsation artifacts are a major causes of difficult-to-assess 3D-visualizations in which the NVC is obscured (see Fig. 6). For the flow artifacts, the presented fusion approach immediately resulted in improved 3D vascular representations (see Table 2), while the pulsation artifacts could be effectively eliminated after optimizing the segmentation of the fused data (see Table 2). Vessels such as PICA or AICA, which were interrupted or completely extinguished in 3D-vis-CISS due to contour fusion, could be visualized significantly better in 3D-vis-FUSION. By optimizing the segmentation of the fused data, the effect was eliminated in almost all cases (see Table 2).

There were also limitations in our study. Although the evaluation of image data is straightforward and easy to understand, the semi-automatic image processing requires expert knowledge about the complex anatomy of the neurovascular relationship needed. In the future, further automation is necessary.

As a further limitation of the presented work, the analysis of image data focuses on arteries. However, it is important to also consider veins as a possible cause of NVC. In the study of Dumot et al., compression veins found in MVD were precisely described and their relevance as a source of TN was shown [5]. Since our approach aims at further automated processing of the image data, it cannot be used safely for veins due to the currently uncertain differentiability of arteries and veins in T2 and T1 data. It will be necessary in the future to also integrate veins into a safe 3D visualization within our framework.

Finally, the classification of the NVC should be analyzed in more detail. In this context, Brinzeu et al. quantitatively examined the predictability of NVC in TN based on protocolized MR data and findings of MVD revealing high prediction values for high grades of compression and lower values with possible false positives in low-grade compression [3]. With regard to classification, we have used color-encoded distance information to calculate the vessel-nerve contact area and to analyze the probability of its extent in an earlier work [20].

## Conclusion

With the presented approach for fusion, the 3D representation of the anatomical course of the vessels and nerves in the posterior fossa is significantly improved. Typical artifacts that affect the assessment of NVC are effectively eliminated. This allows for clearer and more complete 3D visualizations, which are particularly advantageous when using image data at higher field strengths.

In summary, we present an efficient and robust fusion strategy that further enhances our established technique for 3D visualization of NVC. It makes the detection and analysis of NVC syndromes more precise and effectively supports microvascular decompression.

## Data Availability

The datasets generated and/or analyzed during the current study are available from the corresponding author on reasonable request. All raw data and processed image data are stored on servers of the University Hospital of Erlangen.
